# Effects of Exogenous Spermidine on Germination and Seedling Growth of Rice Under Salt Stress: Physiological and Transcriptomic Insights

**DOI:** 10.3390/cimb47110946

**Published:** 2025-11-13

**Authors:** Biaoxin Fei, Jian Liu, Baolai Mao, Ruixiang Wang, Yifan Meng, Haidong Huang, Xin Lu, Fei Zhao, Yongbo Duan

**Affiliations:** 1 College of Agronomy & Resources and Environment, Tianjin Agricultural University, Tianjin 300392, China; feibiaoxin@163.com (B.F.); 13821757926@163.com (J.L.); 15398706882@163.com (B.M.); 15373741420@163.com (R.W.); 17860295645@163.com (Y.M.); hdhuang@tjau.edu.cn (H.H.); luqingxin@126.com (X.L.); 2 Anhui Provincial Engineering Laboratory for Efficient Utilization of Featured Resource Plants, College of Life Sciences, Huaibei Normal University, Huaibei 235026, China

**Keywords:** rice, spermidine, salt stress, transcriptome, physiological biochemistry

## Abstract

Salt stress severely impairs rice (*Oryza sativa* L.) germination and seedling establishment. Exogenous spermidine (Spd) has been shown to regulate stress tolerance in plants, but whether it acts during rice germination and seedling establishment under salt stress remains unclear. Here, rice seeds (cv. Jindao 919) were exposed to 75 mM NaCl with different Spd concentrations (0–1.4 mM), and physiological, biochemical, and transcriptomic responses were evaluated. The findings showed that salt stress had a pronounced inhibitory effect on both seed germination and seedling development. Exogenous Spd effectively alleviated these negative effects, with the most significant improvements observed at 1.0–1.2 mM: germination rate increased by 3.98–8.52%, seedling root length increased by 17.74–37.68%, soluble sugar content increased by 29.83–230%, and SOD and POD activities increased by 29.81–40.3% and 18.45–44.0%, respectively, while MDA content decreased by 36.28–40.3%. Further transcriptomic analysis identified a total of 1835 differentially expressed genes (DEGs). KEGG enrichment analysis revealed these genes were concentrated in key pathways including terpenoid biosynthesis, phenylpropanoid biosynthesis, and amino sugar and nucleotide sugar metabolism, thus alleviating the negative impact of salt stress on rice germination and seedling development. These pathways are closely related to gibberellin metabolism, lignin biosynthesis, and amino sugar metabolism, further revealing the regulatory role of Spd. Overall, 1.0–1.2 mM Spd was most effective in alleviating salt stress by synergistically regulating antioxidant defense, osmoregulation, and metabolic reprogramming, enhancing rice’s overall stress tolerance. This study provides theoretical guidance for precise regulation of Spd concentration to improve rice performance in saline-alkaline soils, and reveals the sustained promoting effects of Spd across various developmental stages of rice and its underlying molecular mechanisms.

## 1. Introduction

Soil salinization is a significant environmental stressor that limits agricultural growth worldwide [1]. It is estimated that over 20% of irrigated agricultural land across the globe is impacted by soil salinity [2]. Salt stress harms plants by causing osmotic imbalance, ion toxicity, nutrient shortages, and oxidative damage due to excess reactive oxygen species (ROS) [3,4,5]. Under short-term stress conditions, plants can mitigate ROS toxicity by enhancing antioxidant enzyme activity to maintain cellular homeostasis. However, when the salt concentration exceeds the tolerance threshold, the antioxidant system collapses, resulting in severe membrane lipid peroxidation, chloroplast degradation, and metabolic disorders, ultimately leading to growth inhibition or even plant death [6]. These effects greatly impair plant development and crop yields in salinized areas. With the challenges posed by rapid population growth and scarce arable land, making effective use of saline soils has become crucial for maintaining food security and promoting sustainable land use. As a major staple crop, rice (*Oryza sativa* L.) holds a central place in China’s efforts to ensure food security [7,8]. However, rice is highly sensitive to salinity [9,10], particularly during germination and seedling establishment, two of the most vulnerable stages for yield formation. Salt stress results in stunted growth, leaf chlorosis, and even plant death [11,12]. Enhancing the salt tolerance of rice is crucial for the sustainable utilization of saline-alkali soils and the maintenance of food security.

Current strategies for improving crop salt tolerance include varietal improvement [13], soil improvement [14], and exogenous substance regulation [15]. Nitrogen fertilizer, widely used to enhance crop productivity, has complex and environmentally dependent effects on saline soils [16,17,18]. To improve salt tolerance and promote plant adaptation under high-salinity conditions, increasing attention has been given to the role of exogenous regulators. For example, foliar application of brassinolide (EBR) significantly enhanced antioxidant enzyme activity in eucalyptus [19], and foliar application of kinetin (KT) reduced lipoxygenase (LOX) activity in loofah, thereby improving its antioxidant capacity [20]. Li et al. [21] reported that exogenous melatonin (MT) treatment reduced Na^+^ and Cl^−^ contents in two-colored lemon, and Al-Mushhin et al. [22] found that foliar inositol application in quinoa scavenged H_2_O_2,_ reduced ROS accumulation, and alleviated the detrimental effects of salinity. Exogenous regulators confer notable advantages by lowering Na^+^ concentrations, facilitating ion sequestration, and promoting the synthesis of osmotic adjustment compounds [23], and thus represent an effective approach to improving plant salt tolerance.

Polyamines (PAs) are small aliphatic nitrogenous compounds ubiquitous in living organisms; in plants, the predominant forms are putrescine (Put), spermidine (Spd), and spermine (Spm) [24]. These PAs play critical roles in growth, development, and stress responses [25,26]. Under abiotic stress, PAs modulate the expression of genes involved in starch metabolism, photosynthesis, antioxidant defense, and stress-signaling pathways [24,25]. Among them, Spd and Spm are particularly implicated in stress adaptation; in rice, elevated endogenous Spd/Spm levels are hallmarks of acclimation to salt stress [27], and exogenous Spd enhances salt tolerance in cucumber in part by promoting reactive oxygen species (ROS) scavenging [28]. In rice roots, transcriptomic studies have begun to elucidate the mechanisms of Spd-mediated salt tolerance: Saha et al. identified Spd-responsive genes associated with antioxidant defense, transport, and signaling [29]. More recently, a complementary study reported that exogenous Spd modulates the expression of high-affinity K^+^ transporter (*OsHAK)* genes in rice roots under salinity, consistent with reprogramming of ion-transport and stress-signaling pathways [30]. In other crop species (e.g., cucumber and tomato), Spd- or polyamine-responsive transcriptome/expression analyses under salinity likewise implicate antioxidant defenses, ion transport, and hormone-related signaling [24,25,28]. Nevertheless, most existing studies have focused on a single organ or developmental stage (predominantly roots) and/or emphasized physiological observations rather than stage-resolved transcriptomes [24,25,29,30]; consequently, the effects of exogenous Spd during seed germination and early seedling development in rice-and the accompanying gene-regulatory programs-remain insufficiently characterized.

To address these limitations, we used the rice cultivar ‘Jindao 919’ to investigate seed germination, seedling growth, physiological traits, and transcriptomic changes under salt stress, with the aim of elucidating the physiological mechanisms and gene regulatory networks mediated by exogenous Spd in rice seedlings in response to salinity. This approach provides a more comprehensive understanding of how Spd enhances rice adaptation to saline environments.

## 2. Materials and Methods

### 2.1. Test Materials

The experiment took place in a climate-controlled growth chamber at Tianjin Agricultural University (38.96° N, 117.12° E) between September and December 2024. The chamber (Nanjing Lithgow Instrument and Equipment Co., Ltd., Nanjing, China) was maintained at 26 °C and 75% relative humidity under a 12 h light/12 h dark photoperiod provided by LED lighting at 150 μmol m^−2^ s^−1^ PPFD. Spatial light uniformity exceeded 90%, with an edge-to-center intensity drop < 15%. These environmental settings (temperature, humidity, and photoperiod) were used for both the germination assay and the young-plant hydroponic assay. The rice cultivar used was ‘Jindao 919’, a japonica conventional rice variety belonging to the well-known Xiaozhan rice group of Tianjin, China. It is a representative local cultivar noted for its excellent grain quality and stable yield performance, widely cultivated in the Tianjin region. Previous regional trials have shown that ‘Jindao 919’ displays a typical japonica-type response to salinity, making it suitable for studying physiological and transcriptomic changes under salt stress. Hydroponic culture was carried out using Hoagland nutrient solution (2.185 g/L). Spermidine (Spd) with purity ≥98% was used, and all other reagents were of analytical grade.

### 2.2. Experimental Design

After surface sterilization with 75% (*v*/*v*) ethanol for 2–3 min, rice seeds were rinsed three times with sterile distilled water and then soaked in Spd solutions of different concentrations (0.6–1.4 mM) for 24 h. Seeds soaked in distilled water and subsequently exposed to 75 mM NaCl were designated as the S0 treatment. Spd immersion solutions (0.6–1.4 mM) and 75 mM NaCl solutions were prepared using sterile distilled water and adjusted to pH 7.0 ± 0.1 at 25 °C using 0.1 M hydrochloric acid or sodium hydroxide. The salinity of Tianjin’s saline–alkali soils typically ranges from 2‰ to 4‰. Therefore, 75 mM NaCl was selected to simulate moderate salt stress, which significantly inhibits rice germination and seedling growth but is not lethal. This concentration effectively allows evaluation of salt tolerance and the alleviating effects of Spd treatment under controlled conditions. Treatments were coded as CK (control, 0 mM NaCl and no spermidine), S0 (75 mM NaCl only), and S1–S5 (75 mM NaCl plus spermidine at 0.6, 0.8, 1.0, 1.2, and 1.4 mM, respectively)

(1) Germination assay: Germination was assessed according to GB/T 3543.4-1995 [31]. Each germination box (12 cm × 12 cm) was lined with two sheets of filter paper and filled with 10 mL of 75 mM NaCl solution. Pretreated seeds were placed in the boxes, 50 per box, ensuring no contact between seeds. Each treatment was replicated three times. Moisture was maintained by adding NaCl solution as needed. Germination was monitored daily, and measurements were taken 14 days after sowing.
(2) Young plant assay: Pretreated seeds were first grown in seedling trays (60 cm × 30 cm × 3 cm) to the three-leaf stage, then transferred to a hydroponic system containing half-strength Hoagland solution with 75 mM NaCl. Plants were set in hydroponic boxes (125 cm × 85 cm × 115 cm, 6 mm holes) with five plants per hole, secured by cotton plugs. Each treatment had three biological replicates. After 10 days of salt stress, seedlings were collected and stored at −80 °C for physiological and biochemical analysis.

### 2.3. Measurement Items and Methods

#### 2.3.1. Determination of Relevant Indicators in Rice Germination Tests

In accordance with the GB/T 3543.4-1995 [31] standard, several parameters were determined, including germination potential (GP), germination rate (GR), germination index (GI), and vitality index (VI). For phenotypic assessment, ten uniform seedlings per treatment were randomly selected. After blotting surface moisture, seedling length (SL) and seedling root length (SRL) were measured with a ruler. Fresh weights of aboveground (AFW) and belowground (BFW) tissues were determined using a precision balance (0.001 g accuracy). Samples were oven-dried at 105 °C for 30 min, followed by drying at 80 °C for 72 h, and the dry weights of aboveground (ADW) and belowground (BDW) parts were recorded. For each treatment, seedlings showing severe wilting, necrosis, or abnormal development unrelated to treatment effects were excluded prior to measurement. Uniform and healthy seedlings with consistent growth status were selected for sampling and subsequent physiological and morphological measurements to ensure data reliability.

#### 2.3.2. Measurement of Relevant Indicators in Rice Seedling Experiments

During the seedling experiment, rice leaves were randomly cut using scissors, and the samples were promptly frozen in liquid nitrogen for later physiological analysis. Plant height (PH), root length (RL), shoot fresh weight (SFW), and root fresh weight (RFW) were measured according to the procedures outlined in Section 2.3.1.

Physiological and biochemical parameters were determined as follows: superoxide dismutase (SOD) activity was measured using the nitroblue tetrazolium photoreduction method [32]; peroxidase (POD) activity was assessed by the guaiacol colorimetric method [33]; catalase (CAT) activity was determined according to the method described in [34]; and malondialdehyde (MDA) levels were measured by the thiobarbituric acid (TBA) colorimetric assay [35]. Soluble protein content was measured using the Coomassie Brilliant Blue G250 method [36]. Hydrogen peroxide (H_2_O_2_), superoxide anion (O^2−^), and soluble sugar contents were determined using commercial kits (Solarbio, BC3595; Solarbio, BC1295; Solarbio, BC0035, Beijing, China), respectively.

### 2.4. Transcriptome Sequencing and Data Analysis

Based on a preliminary comprehensive salt tolerance assessment constructed using the membership function approach, three treatment groups were selected for transcriptome sequencing and qRT-PCR analysis: the salt stress control group (S0), the low-concentration Spd treatment group (S1), and the high-concentration Spd treatment group (S4). Fresh leaves from each group were promptly frozen in liquid nitrogen, and total RNA was extracted using the Polysaccharide and Polyphenol Plant Total RNA Extraction Kit (TIANGEN). After assessing RNA quality, transcriptome libraries were prepared and sequenced by Shanghai Paisenno Company using second-generation sequencing technology (Next-Generation Sequencing, NGS) on the Illumina platform with paired-end (PE) sequencing.

High-quality clean reads were obtained by filtering raw data and subsequently mapped to the rice reference genome (*Oryza_sativa*.IRGSP-1.0.dna.toplevel.fa), with mapping rates between 97.91% and 98.52%. Differential gene expression was analyzed using DESeq2 (v1.38.3). Raw Wald-test *p*-values were adjusted for multiple testing using the Benjamini–Hochberg false discovery rate (FDR) procedure as implemented in DESeq2, and genes with |log2FoldChange| > 1 and adjusted *p*-value (padj, FDR) < 0.05 were considered significant. Analyses including differential expression, gene annotation, GO and KEGG enrichment, and pathway analysis were performed using the Paishenno Gene Cloud Platform (https://www.genescloud.cn, accessed on 25 August 2025).

Transcriptome results were validated by reverse transcribing RNA to cDNA with the Genesand Biotech SR511 kit, followed by qRT-PCR using the SQ410 kit on an ABI 7500 system. Eight DEGs were randomly selected for validation, with gene-specific primers designed via NCBI Primer-BLAST. OsActin served as the internal control, and relative expression was calculated using the 2^−ΔΔC^ method. Primer sequences and PCR conditions are detailed in Appendix A Table A1.

### 2.5. Statistical Analysis

Salt tolerance evaluation was performed using membership functions and principal component analysis (PCA) [37], as follows:

(1) Data reduction: PCA was applied to extract principal components with cumulative eigenvalues explaining more than 85% of the variance.
(2) Data standardization: Based on the principles of fuzzy mathematics, membership functions were used to normalize the scores of each principal component. The membership function formulas were as follows:

For indicators positively correlated with salt tolerance:

(1) $$\mu (Xij)=\frac{Xij-X\min}{X\max-X\min}$$

For indicators negatively correlated with salt tolerance:

(2) $$\mu (Xij)=\frac{X\max-Xij}{X\max-X\min}$$

(3) Comprehensive evaluation: The weight (*W*_j_) of each principal component was determined using its cumulative variance contribution rate. The D value was then calculated using the following formula:

(3) D=∑(Xij×Wj)

Here, *X*_ij_ is the score of the jth principal component for the ith sample, with *X*_min_ and *X*_max_ as the respective minimum and maximum values. The *D* represents the overall salt tolerance index. Data were processed in Microsoft Excel 2019 (Microsoft Corporation, Redmond, WA, USA). Statistical analysis was done using one-way ANOVA and Duncan’s test in IBM SPSS Statistics 26. Figures were created using GraphPad Prism 9.5.0 (GraphPad Software, San Diego, CA, USA), Origin 2024 (OriginLab Corporation, Northampton, MA, USA), and other relevant software.

## 3. Results

### 3.1. Effects of Exogenous Spermidine on Rice Seed Viability Under Salt Stress

Rice seeds exposed to salt stress showed markedly reduced viability, and in severe cases germination was completely inhibited, preventing seedling establishment. As shown in Table 1, compared with the control (CK), the germination potential and germination rate decreased significantly by 7.50% and 8.0%, respectively. Similarly, the vigor index and germination index, which reflect germination capacity and speed, were reduced by 33.78% and 27.85%, respectively. These results demonstrate that salt stress substantially impairs seed germination. Exogenous Spd treatment (S1–S5) effectively alleviated these inhibitory effects, as indicated by improvements in germination potential, germination rate, and related indices across all treatment groups. Among them, the S4 treatment produced the most pronounced effect, with germination potential (93%), germination rate (95.5%), vigor index (130.02), and germination index (15.66) reaching their highest values. Compared with S0, these increases corresponded to 5.68%, 8.52%, 46.0%, and 29.96%, respectively. Notably, although S5 treatment still showed a positive effect relative to S0, all germination parameters were significantly lower than those observed under S4. This suggests that excessively high Spd concentrations weaken the promotive effect, indicating the existence of an optimal concentration range. These findings imply that Spd not only restores germination ability under stress but also contributes to improved early seedling development.

### 3.2. Effects of Exogenous Spermidine on Rice Germination Morphology Under Salt Stress

As shown in Table 2, salt stress (S0) markedly inhibited seedling morphological traits and biomass accumulation. Compared with CK, shoot length, root length, and root number decreased significantly by 9.87%, 24.30%, and 24.08%, respectively. Similarly, aboveground fresh weight, belowground fresh weight, aboveground dry weight, and belowground dry weight decreased by 13.32%, 11.25%, 11.23%, and 13.04%, respectively. Seed soaking with exogenous Spd (S1–S5) alleviated these inhibitory effects to varying degrees. Under S4, seedling shoot length (8.31 cm), root length (9.39 cm), and root number (6.03) showed notable improvements compared to S0. Relative to S0, these parameters increased by 12.44%, 37.68%, and 27.48%, respectively. Biomass accumulation was also higher under S4, with aboveground fresh weight (30.21 mg), belowground fresh weight (56.99 mg), and belowground dry weight (13.75 mg) showing increases of 17.87%, 14.88%, 20.57%, and 22.77% compared with S0. These results suggest that S4 treatment effectively alleviated salt-induced damage and promoted seedling growth, particularly in root traits. However, excessively high Spd concentrations (S5) were less effective: although S5 still improved growth compared with S0, its effects were weaker than those of S4, indicating the existence of an optimal concentration range. The improvements in root length and root number under S4 suggest that Spd may preferentially regulate root developmental programs, which are crucial for nutrient uptake and stress adaptation during early establishment.

### 3.3. Effects of Exogenous Spermidine on the Agronomic Traits of Rice Young Plants Under Salt Stress

As shown in Table 3, results from the young plant assay demonstrated that salt stress (S0) significantly suppressed rice seedling growth compared to the control (CK), resulting in decreases of 24.29% in PH and 27.61% in RL. Biomass parameters were also significantly affected: SFW, SDW, rRFW, and RDW decreased by 33.96%, 29.98%, 42.10%, and 24.06%, respectively. Exogenous Spd treatments (S1–S4) alleviated these inhibitory effects to varying extents. Compared with S0, PH increased by 9.03–16.6%, RL by 3.47–46.46%, SFW by 19.56–52.62%, SDW by 13.87–39.33%, and RFW by 43.54–80.56%. The most pronounced growth-promoting effects were observed under the S3 and S4 treatments. However, it is noteworthy that the promotive effect of Spd was not linearly related to concentration. At excessively high Spd levels (S5), growth was suppressed: PH decreased by 4.84%, RL by 0.42%, and SFW by 3.82% compared with S4. These results suggest that there is an optimal concentration range of Spd for enhancing seedling growth. Additionally, the increase in root biomass was particularly notable, suggesting that Spd may reinforce root system adaptability, which could be essential for strengthening the plant’s overall salt tolerance.

### 3.4. Effects of Exogenous Spermidine on the Physiological and Biochemical Characteristics of Rice Young Plants Under Salt Stress

The young plant assay was further employed to explore the physiological basis of spermidine-induced salt tolerance. Specifically, we assessed changes in antioxidant enzyme activities, osmotic regulatory compounds, and oxidative stress markers to clarify the functional role of spermidine in maintaining redox equilibrium and osmotic adjustment under salinity.

#### 3.4.1. Effects of Exogenous Spermidine on the Antioxidant Enzyme System of Rice Young Plants Under Salt Stress

**Figure 1:**
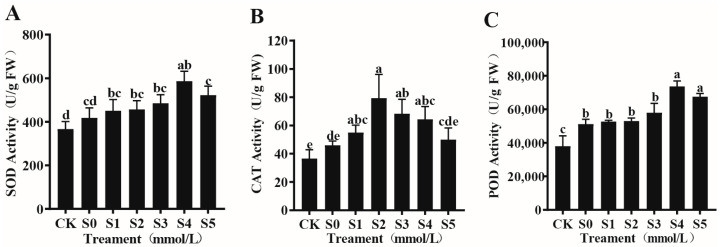
Effects of exogenous Spd on SOD (**A**), CAT (**B**), and POD (**C**) activities in rice young plants under salt stress. Data are expressed as mean ± SD (*n* = 3). Distinct lowercase letters denote statistically significant differences (*p* < 0.05) based on one-way ANOVA and Duncan’s multiple range test. Treatments-CK: control (no NaCl, no spermidine); S0: 75 mM NaCl only; S1–S5: 75 mM NaCl + spermidine at 0.6, 0.8, 1.0, 1.2, and 1.4 mM, respectively.

As shown in Figure 1A–C, the activities of rice antioxidant enzymes (SOD, POD, and CAT) followed a typical bell-shaped pattern, increasing initially and then declining with higher Spd concentrations. Compared with CK, S0 treatment significantly enhanced antioxidant enzyme activity, with SOD, POD, and CAT increasing by 28.6%, 31.2%, and 41.3%, respectively. This indicates that salt stress activates the antioxidant defense system of rice to counteract excessive ROS accumulation. Exogenous Spd (S1–S5) further stimulated antioxidant enzyme activities. The maximum activities of SOD (586.60 U/g FW) and POD (73,666.67 U/g FW) were observed under the S4 treatment, representing increases of 40.30% and 43.97% compared with S0. CAT activity peaked at 79.33 U/g FW under the S2 treatment, corresponding to a 72.46% increase over S0. Although SOD, POD, and CAT activities under S5 were still 18.2%, 21.5%, and 35.8% higher than S0, their levels were significantly lower than those under S4, suggesting that excessively high Spd concentrations attenuate the promotive effect. This bell-shaped response indicates that Spd acts not only as a passive antioxidant enhancer but also as a fine-tuned regulator of redox homeostasis, actively triggering defense signaling pathways under moderate concentrations.

#### 3.4.2. Effects of Exogenous Spermidine on Osmotic Regulatory Substances in Rice Young Plants Under Salt Stress

**Figure 2:**
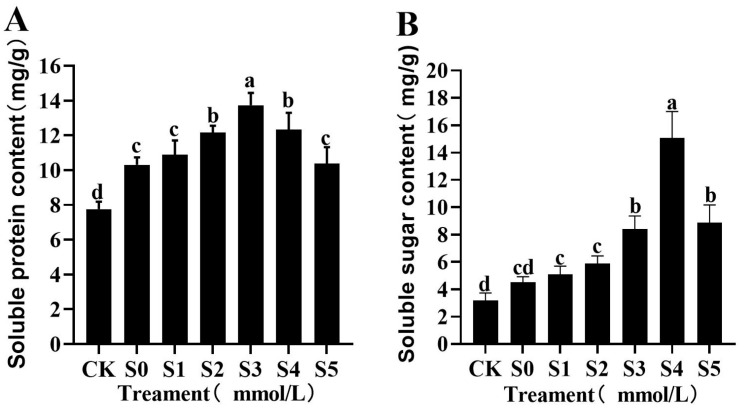
Effects of exogenous Spd on soluble protein (**A**) and soluble sugar (**B**) contents in rice young plants under salt stress. Data are expressed as mean ± SD (*n* = 3). Distinct lowercase letters denote statistically significant differences (*p* < 0.05) based on one-way ANOVA and Duncan’s multiple range test. Treatment codes as defined in Figure 1 legend.

Salt stress promoted the buildup of osmotic adjustment compounds in rice young plants (Figure 2A,B). Both soluble protein and soluble sugar contents displayed a bell-shaped trend, first increasing and then decreasing with higher Spd concentrations. Under S0 treatment, soluble protein (10.30 mg/g) and soluble sugar (4.52 mg/g) increased significantly by 32.90% and 41.69%, respectively, compared with CK. Exogenous Spd treatments (S1–S5) further enhanced the accumulation of these substances to varying degrees. Soluble protein content peaked at 13.72 mg/g under S3, representing a 33.20% increase over S0. Soluble sugar content reached its maximum of 15.08 mg/g under S4, a striking 230% increase relative to S0. Although soluble protein under S5 was still higher than S0, it was significantly lower than that of S3. Similarly, soluble sugar under S5 (8.88 mg/g) increased by 96.46% compared with S0 but was 58.78% lower than the S4 level. The striking increase in soluble sugar under S4 suggests that Spd-driven sugar metabolism may serve dual roles as osmoprotectants and as energy/signaling molecules supporting growth recovery under salinity.

#### 3.4.3. Effects of Exogenous Spermidine on Oxidative Stress in Rice Young Plants Under Salt Stress

**Figure 3:**
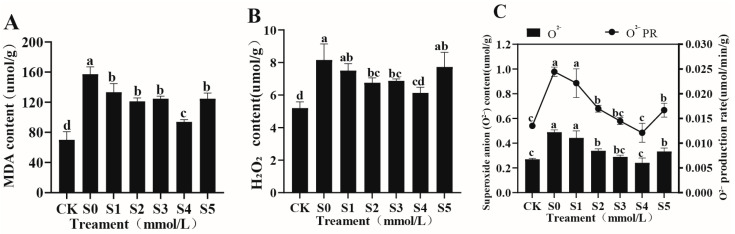
Effects of exogenous Spd on oxidative stress indicators in rice young plants under salt stress: (**A**) MDA content, (**B**) H_2_O_2_ content, (**C**) superoxide anion content and production rate. Data are expressed as mean ± SD (*n* = 3). Distinct lowercase letters denote statistically significant differences (*p* < 0.05) based on one-way ANOVA and Duncan’s multiple range test. Treatment codes as defined in Figure 1 legend.

As shown in Figure 3A–C. Under S0 treatment, the levels of MDA (157.19 μmol/g), H_2_O_2_ (8.16 μmol/g), O^2−^ (0.4889 μmol/g), and O^2−^ production rate (0.0244 μmol/min/g) reached their peak, representing increases of 124.05%, 56.92%, 81.21%, and 78.52%, respectively, compared with CK. Exogenous Spd effectively mitigated these effects. Under S4 treatment, all four indicators dropped to their lowest values (93.84 μmol/g, 6.13 μmol/g, 0.2415 μmol/g, and 0.0121 μmol/min/g), corresponding to reductions of 24.88–50.60% relative to S0. By contrast, under the high-concentration S5 treatment, all indicators rebounded significantly, suggesting the presence of a dose-dependent threshold effect. The sharp reduction in MDA and ROS levels under S4 implies that Spd effectively reinforces membrane stability and suppresses oxidative injury, creating a cellular environment conducive to sustained growth.

### 3.5. Comprehensive Analysis of the Effects of Exogenous Spermidine on the Growth and Physiology of Rice Young Plants Under Salt Stress

To further evaluate the overall effects of spermidine treatment at the young plant stage, all growth, physiological, and biochemical parameters obtained from the young plant assay were subjected to integrated statistical analysis. Principal component analysis (PCA) and subordinate function analysis were performed to comprehensively assess the contribution of different indicators to salt tolerance and to identify the most effective spermidine concentration. These analyses provided a quantitative basis for selecting representative treatments (S0, S1, and S4) for subsequent transcriptome sequencing.

#### 3.5.1. Principal Component Analysis

**Figure 4:**
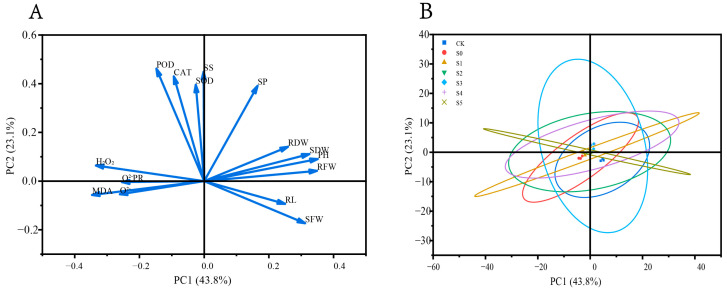
Principal component analysis of physiological and biochemical indices of rice young plants under different Spd treatments. (**A**) Environmental Factor Load Chart, (**B**) Sample Principal Component Distribution Ellipsoid Diagram.

Principal component analysis (PCA) was performed on the growth and physiological-biochemical parameters of rice young plants exposed to various concentrations of exogenous Spd. As shown in Table 4, five principal components with eigenvalues >1 were extracted, explaining a cumulative variance of 88.32%. Among them, PC1 had an eigenvalue of 6.57, accounting for 43.81% of the total variance, while PC2 had an eigenvalue of 3.47, explaining 23.11%. The loading plot (Figure 4A) indicated that PC1 was mainly associated with PH, RFW, SDW, SFW, and RDW, whereas PC2 was primarily associated with POD, SS, CAT, SOD, and SP. These variables collectively represent the key traits contributing to salt tolerance in rice seedlings.

#### 3.5.2. Subordinate Function Analysis

Using principal component analysis (PCA), we reduced the dimensionality of all young plants parameters. The principal components identified in Section 3.5.1 were treated as composite indicators and standardized via membership functions. For each composite indicator, a weight (*W*_j_) was assigned in proportion to its variance contribution to the cumulative variance of the corresponding principal component (Table 5). The comprehensive evaluation value (D) was then calculated as the weighted sum of the standardized indicators. The D value was lowest under salt stress alone (S0). With increasing concentrations of exogenous Spd (S1–S4), D showed an overall upward trend, indicating enhanced comprehensive stress tolerance in rice seedlings. The highest D values occurred under S3–S4 and were significantly greater than CK, supporting that 1.0–1.2 mM Spd exerts the most pronounced growth-promoting effect under salinity. This integrated assessment suggests that Spd-mediated improvements reflect coordinated regulation of growth, redox homeostasis, and osmotic adjustment, and it guided the selection of S0, S1, and S4 for subsequent RNA−seq analysis.

### 3.6. Transcriptome Analysis of the Effects of Exogenous Spermidine on the Growth of Rice Young Plants Under Salt Stress

Following the comprehensive evaluation of the young plant assay (Section 3.5), leaves from salt-stressed control (S0), low−Spd (S1), and high-Spd (S4) treatments were collected from young plants for RNA sequencing. This section therefore reports transcriptomic changes derived specifically from the young plant assay, providing molecular context for the growth and physiological responses described above.

#### 3.6.1. Differential Gene Expression Analysis and Enrichment Analysis

**Figure 5:**
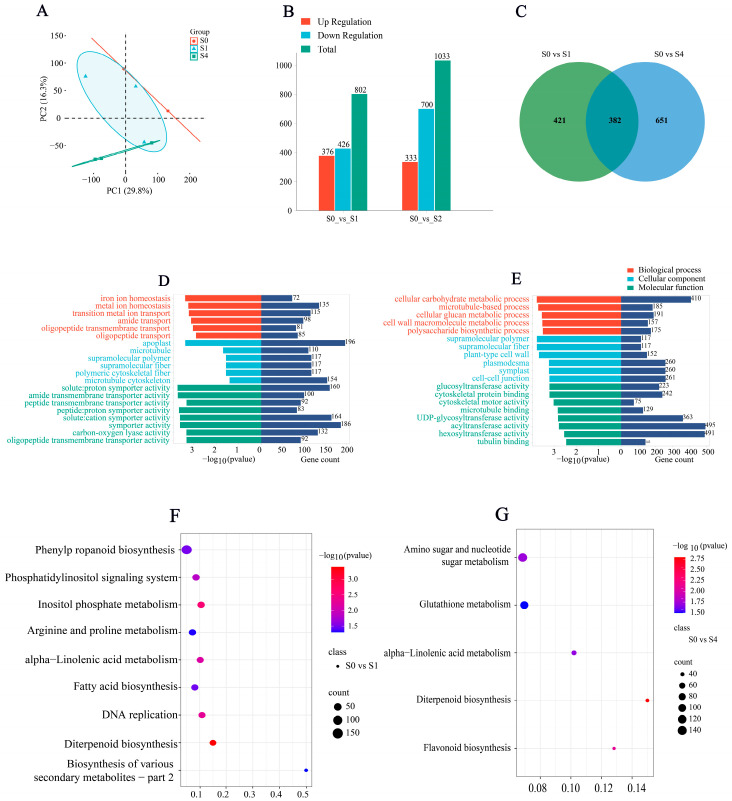
Transcriptome analysis of rice young plants under salt stress with different Spd concentrations: (**A**) PCA, (**B**) number of DEGs in S0 vs. S1, (**C**) Venn diagram, (**D**) GO enrichment (S0 vs. S1), (**E**) GO enrichment (S0 vs. S4), (**F**) KEGG enrichment (S0 vs. S1), and (**G**) KEGG enrichment (S0 vs. S4). For RNA-Seq: S0 = 75 mM NaCl; S1 = 75 mM NaCl + 0.6 mM spermidine; S4 = 75 mM NaCl + 1.2 mM spermidine.

In leaves collected from the young plant assay, we generated 384,706,334 raw reads across nine libraries; after trimming adapters and removing low-quality reads, 379,922,196 clean reads remained. All libraries exhibited high sequencing quality (Q30 > 96%, GC: 49–50%) and mapping efficiencies of 97.9–98.5% to the rice reference genome, indicating excellent data integrity (Appendix A Table A2). These quality metrics confirm that the RNA-seq data were suitable for subsequent differential expression and enrichment analyses. Reads mapped to the rice reference genome at high rates (97.91–98.52%; Methods 2.4). Principal component analysis (Figure 5A) showed tight clustering of biological replicates, indicating excellent reproducibility. As shown in Figure 5B,C, differential expression analysis identified 802 and 1033 DEGs in the S0 vs. S1 and S0 vs. S4 comparisons, respectively, with 382 shared; in S0 vs. S1, 376 genes were up-regulated and 426 down-regulated, and in S0 vs. S4, 333 were up-regulated and 700 down-regulated. GO annotation assigned 2566 and 2535 DEGs to categories such as metal ion homeostasis, symporter activity, carbon–oxygen lyase activity, microtubule cytoskeleton, lyase activity, manganese ion transport, and plastid (Figure 5D,E). KEGG enrichment highlighted phenylpropanoid biosynthesis, phosphatidylinositol signaling, diterpenoid biosynthesis, and α-linolenic acid metabolism in S0 vs. S1, and amino sugar and nucleotide sugar metabolism, glutathione metabolism, α-linolenic acid metabolism, and diterpenoid biosynthesis in S0 vs. S4 (Figure 5F,G), supporting spermidine-mediated regulation of hormone metabolism, lignin biosynthesis, and carbon allocation under salt stress.

#### 3.6.2. Differential Gene Expression Patterns

**Figure 6:**
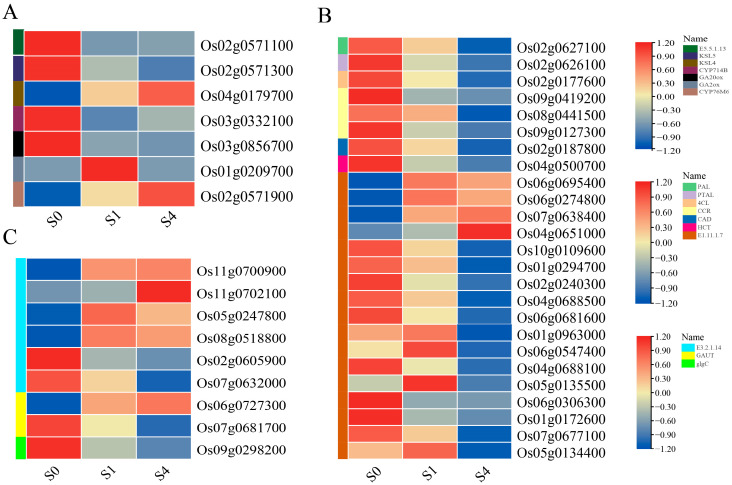
KEGG pathway enrichment of DEGs in rice young plants: (**A**) diterpenoid biosynthesis, (**B**) phenylpropanoid biosynthesis, and (**C**) amino sugar and nucleotide sugar metabolism. Note: Colored boxes on the right side represent the corresponding enzyme names involved in each enriched pathway. For RNA−Seq: S0 = 75 mM NaCl; S1 = 75 mM NaCl + 0.6 mM spermidine; S4 = 75 mM NaCl + 1.2 mM spermidine. The color intensity reflects the relative expression level (log_2_ fold change) under each treatment.

In the KEGG enrichment analysis, diterpenoid biosynthesis (map00904) was significantly enriched in both the S0 vs. S1 and S0 vs. S4 comparisons, suggesting that this pathway may be involved in the transcriptional response to exogenous Spd under salt stress (Figure 6A). Compared with the S0 treatment, expression of Os01g0209700 (GA2ox) was upregulated in S1, while GA20ox and CYP714B showed decreased expression. Under the high-concentration S4 treatment, KSL4 and CYP76M6 were upregulated, whereas CYP714B, E5.5.1.13, and KSL5 were downregulated. These transcriptional changes suggest that diterpenoid-related genes respond to Spd treatment, and their expression patterns may be linked to gibberellin (GA) metabolic processes. However, since hormone quantification was not performed, this association remains correlative and requires further biochemical validation.

In the phenylpropanoid biosynthesis pathway (map00940), several key protein families were significantly enriched (Figure 6B). Among them, genes encoding the rate-limiting enzyme CCR (Os09g0419200, Os08g0441500, Os09g0127300) were downregulated. Multiple peroxidase genes (E1.11.1.7) also showed differential expression, with some upregulated and others downregulated between S0 and S4. These findings imply that Spd treatment may influence phenylpropanoid-related metabolic adjustments under salt stress, but the functional relevance to lignin biosynthesis needs to be experimentally verified.

The amino sugar and nucleotide sugar metabolism pathway (map00520) was specifically enriched under S4 treatment (Figure 6C). A total of nine DEGs were mapped to this pathway, including genes associated with E3.2.1.14 and GAUT family proteins. These genes participate in UDP−glucose conversion and chitin recycling, processes that may support cell-wall formation and osmotic balance during salt stress. Nevertheless, without direct measurement of soluble or structural sugar content, the link between gene activation and enhanced stress tolerance remains hypothetical.

Overall, the transcriptomic results indicate that Spd treatment is associated with altered expression of genes in diterpenoid, phenylpropanoid, and amino sugar metabolic pathways. These observations highlight potential molecular targets for Spd-mediated stress responses but should be interpreted as correlations rather than causal mechanisms pending further physiological and genetic validation.

#### 3.6.3. qRT-PCR Validation

**Figure 7:**
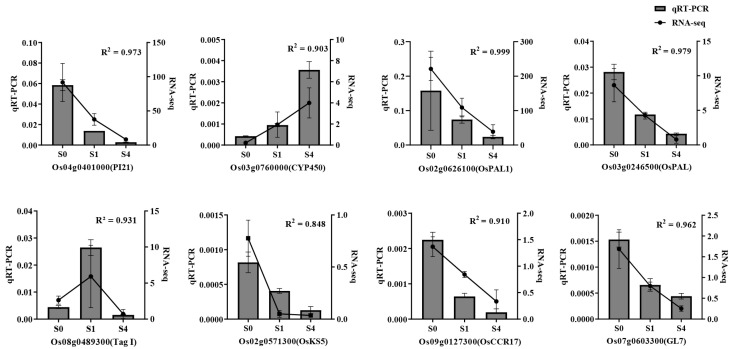
qRT-PCR validation of eight selected DEGs. Expression patterns obtained by RNA-Seq and qRT-PCR showed consistent trends. Values are means ± SD (*n* = 3). The genes used for qRT-PCR validation were randomly selected from differentially expressed genes identified in transcriptomic analysis. Their standardized gene names are provided in Table A2. For RNA-Seq: S0 = 75 mM NaCl; S1 = 75 mM NaCl + 0.6 mM spermidine; S4 = 75 mM NaCl + 1.2 mM spermidine.

To further validate the RNA sequencing results, we selected eight key genes for qRT-PCR analysis based on the findings presented in Figure 5 and Figure 6. Potential physiological responses and metabolic regulation during salt stress in rice young plants may involve these genes. The qRT−PCR data, shown in Figure 7, exhibit expression trends consistent with the RNA sequencing results, thereby further corroborating the reliability of the sequencing data.

## 4. Discussion

In the context of increasing global climate change, soil salinization is becoming an increasingly severe issue, not only restricting agricultural productivity but also threatening food security. Unlike previous studies that primarily focused on a single physiological mechanism or transcript level, this study covers two critical stages: seed germination and seedling growth. By integrating transcriptome sequencing, we systematically elucidate the mechanisms by which exogenous Spd regulates salt tolerance in rice under salt stress, providing a more comprehensive perspective on the molecular basis of Spd-enhanced salt adaptation in rice.

Seed germination and seedling growth are key stages in the rice life cycle, where salt tolerance directly impacts subsequent growth, development, and yield formation [38]. The results of this study show that salt stress significantly inhibits germination vigor, vitality index, and seedling biomass, consistent with previous reports on the early effects of salt damage and stage-specific interventions [39]. Spd alleviated salt-induced damage in a concentration-dependent manner, with the most stable comprehensive effects observed in the 1.0–1.2 mM range. At 1.2 mM, in addition to the recovery of growth-related parameters, significant increases in root length, root number, and underground biomass were observed, suggesting that Spd may activate root development regulatory pathways [40,41]. A similar pattern was observed at the seedling stage, where the optimal Spd concentration (1.0–1.2 mM) effectively mitigated the inhibitory effects of salt stress on plant height, root length, and biomass, consistent with studies on the photosynthetic and adaptive regulation of rice under salt stress [42]. When the concentration exceeded 1.2 mM, the promoting effect turned into inhibition, which aligns with the common polyamine “low-dose promotion-high-dose inhibition” rule [43,44]. Similar bell-shaped dose–response patterns of exogenous spermidine have been reported in other plant species, such as white clover and cucumber, where moderate concentrations enhanced antioxidant defense, while excessive spermidine led to metabolic imbalance and ROS accumulation [28,30]. This suggests a threshold mechanism related to spermidine uptake and polyamine catabolism under salt stress. Overall, Spd primarily improves root traits and may enhance whole-plant salt tolerance through root regulation; this conclusion is in agreement with Shen et al.’s findings on “exogenous Spd enhancing rice root salt tolerance” [41].

Abiotic stress typically triggers metabolic reprogramming in plants to reduce cellular damage [45,46,47]. In this study, salt stress led to increased ROS and lipid peroxidation, while the appropriate Spd concentrations increased SOD, POD, and CAT activities, alleviating oxidative damage. At 1.2 mM Spd treatment, soluble sugar levels showed the greatest increase, functioning both as an osmotic protectant and in energy and signal regulation [48,49]; this observation is consistent with reports in crops such as oats and tomatoes [50,51]. Further evidence suggests that Spd’s regulation of carbohydrate metabolism is closely related to the enhancement of rice salt tolerance, beyond the classical antioxidant pathways.

Transcriptome analysis further clarified the molecular mechanisms by which Spd enhances salt tolerance. KEGG enrichment analysis showed that differentially expressed genes were mainly concentrated in key pathways such as diterpene biosynthesis, phenylpropanoid biosynthesis, and amino sugar and nucleotide sugar metabolism. Among the diterpene biosynthesis pathways, genes such as GA2ox, GA20ox, and CYP714B exhibited significant expression changes, suggesting that Spd might balance “growth recovery-stress inhibition” by regulating gibberellin (GA) metabolism [41,52]. Combined with the improvement in root phenotypes, the diterpene pathway is likely involved in regulating root plasticity. Existing studies also suggest that the *OsbZIP73*-mediated enhancement of root salt tolerance is associated with Spd [41]. The *OsEIL1-OsWOX11* module regulates crown root development under soil compaction, demonstrating the synergistic effects of hormone networks in root adaptation [40]. *OsNAC120* integrates growth and resistance by balancing gibberellins and abscisic acid [52]. In the amino sugar and nucleotide sugar metabolism pathways, glucose can broadly reprogram the transcriptional network [48]; in rice, sugar metabolism genes, soluble sugars/starch, and photosynthesis and yield traits are closely related [49]. Multiple results indicate that Spd can regulate non-structural carbohydrates and the activity of key metabolic enzymes [50], and it promotes the recovery of sugar metabolism pathways in aging sorghum seeds, forming a chain of “sugar increase-activation-support recovery” [53]. The changes in genes related to amino sugar and nucleotide sugar metabolism and GAUT (a glycosyltransferase) observed in this study are consistent with the above understanding, indicating that Spd supports stress-induced structural and metabolic repair by optimizing carbon flow distribution. In the phenylpropanoid biosynthesis pathway, the regulation of key enzyme CCR expression suggests that Spd may influence lignin deposition, balancing structural reinforcement and metabolic cost; exogenous glucose promotes the phenylpropanoid pathway and lignin deposition [54], further confirming the close connection between sugar signaling and cell wall remodeling.

Despite the valuable findings of this study, several limitations should be acknowledged. The experiments were performed under controlled conditions using a single rice cultivar, and the transcriptomic results lack functional validation, leaving the field applicability of exogenous Spd uncertain. In addition, representative images of seedlings were not recorded during the experiment, which limits the visual presentation of morphological differences in shoots and roots. Future studies will include photographic documentation to complement the quantitative measurements and better illustrate the phenotypic effects of spermidine under salt stress. Nevertheless, Spd priming shows promise as a practical, low-cost strategy to enhance rice salt tolerance, especially during germination and early growth. Future studies should focus on (1) field-based trials under varying salinity levels and across diverse rice genotypes to assess the robustness of these findings; (2) functional validation of key candidate genes and pathways through targeted genetic and biochemical analyses; and (3) elucidation of the signaling and epigenetic mechanisms underlying Spd-induced stress memory. Such work will deepen our understanding of Spd-mediated salt tolerance mechanisms and provide a theoretical foundation and practical basis for breeding high-salt-tolerant rice varieties adapted to saline–alkali environments.

## 5. Conclusions

This study showed that exogenous spermidine (Spd) alleviates salt-induced inhibition of rice germination and seedling growth, with the optimal effect at 1.0–1.2 mM. At this concentration, Spd markedly improved germination potential, root development, and soluble sugar accumulation. Transcriptomic analyses revealed that Spd treatment was associated with changes in the expression of genes involved in gibberellin metabolism, lignin biosynthesis, and carbon allocation. These correlations suggest that Spd may influence these pathways, thereby contributing to enhanced salt tolerance. Overall, the findings provide transcriptomic evidence supporting Spd’s potential regulatory role in rice salt adaptation and offer a basis for future mechanistic validation.

## Figures and Tables

**Table 1:** The Effect of Exogenous Spermidine on the Viability of Rice Seeds under Salt Stress.

Treatment	Germination Potential (%)	Germination Rate (%)	Vigor Index	Germination Index
CK	95.50 ± 2.52 ^a^	96.00 ± 1.63 ^a^	134.47 ± 9.03 ^a^	16.66 ± 1.23 ^a^
S0	88.00 ± 2.00 ^b^	88.00 ± 2.00 ^c^	89.05 ± 1.59 ^d^	12.05 ± 0.23 ^e^
S1	89.00 ± 2.58 ^b^	89.50 ± 1.92 ^bc^	100.45 ± 11.63 ^bc^	12.62 ± 0.81 ^de^
S2	89.50 ± 3.79 ^ab^	92.50 ± 1.00 ^ab^	107.57 ± 4.64 ^bc^	13.67 ± 0.55 ^cd^
S3	91.50 ± 5.26 ^ab^	94.00 ± 2.83 ^a^	117.62 ± 9.19 ^b^	14.62 ± 0.35 ^bc^
S4	93.00 ± 4.76 ^ab^	95.50 ± 1.00 ^a^	130.02 ± 5.46 ^a^	15.66 ± 0.73 ^ab^
S5	89.50 ± 3.00 ^ab^	90.00 ± 4.00 ^bc^	101.40 ± 6.31 ^c^	13.15 ± 0.88 ^de^

The values in the table are presented as mean ± SD (*n* = 3). Distinct lowercase letters within the same column denote significant differences at *p* < 0.05, while identical letters indicate no significant difference (*p* > 0.05). Treatments-CK: control (no NaCl, no spermidine); S0: 75 mM NaCl only; S1–S5: 75 mM NaCl + spermidine at 0.6, 0.8, 1.0, 1.2, and 1.4 mM, respectively.

**Table 2:** Effects of Exogenous Spermidine on the Germination Morphology of Rice Seeds under Salt Stress.

Treatment	Seedling Length (cm)	Seedling Root Length (cm)	Number of Roots	Aboveground Fresh Weight (mg)	Belowground Fresh Weight (mg)	Aboveground Dry Weight (mg)	Belowground Dry Weight (mg)
CK	8.09 ± 0.67 ^a^	9.01 ± 0.85 ^a^	6.23 ± 0.79 ^a^	29.57 ± 2.44 ^ab^	55.89 ± 2.15 ^a^	6.68 ± 0.49 ^ab^	12.88 ± 0.54 ^a^
S0	7.39 ± 0.10 ^b^	6.82 ± 0.35 ^c^	4.73 ± 0.25 ^c^	25.63 ± 0.65 ^d^	49.61 ± 0.84 ^c^	5.93 ± 0.32 ^b^	11.20 ± 0.46 ^b^
S1	7.94 ± 0.48 ^ab^	6.78 ± 0.45 ^c^	5.65 ± 0.37 ^ab^	26.88 ± 1.64 ^bc^	49.56 ± 0.79 ^c^	6.20 ± 0.95 ^ab^	11.43 ± 0.87 ^b^
S2	7.88 ± 0.32 ^ab^	6.85 ± 0.43 ^c^	5.45 ±0.24 ^abc^	29.02 ± 0.93 ^abc^	51.76 ± 1.11 ^bc^	7.07 ± 0.15 ^a^	13.23 ± 1.02 ^a^
S3	8.04 ± 0.44 ^ab^	8.03 ± 0.55 ^b^	5.73 ± 0.56 ^ab^	29.50 ± 1.69 ^ab^	52.12 ± 2.22 ^b^	7.17 ± 0.35 ^a^	13.23 ± 0.74 ^a^
S4	8.31 ± 0.23 ^a^	9.39 ± 0.62 ^a^	6.03 ± 0.39 ^ab^	30.21 ± 1.52 ^a^	56.99 ± 1.20 ^a^	7.15 ± 0.59 ^a^	13.75 ± 0.62 ^a^
S5	7.72 ± 0.20 ^ab^	7.60 ± 0.89 ^bc^	5.23 ± 0.52 ^bc^	27.24 ± 1.04 ^bcd^	51.84 ± 1.09 ^bc^	5.90 ± 0.46 ^b^	12.85 ± 0.48 ^a^

The values in the table are presented as mean ± SD (*n* = 3). Distinct lowercase letters within the same column denote significant differences at *p* < 0.05, while identical letters indicate no significant difference (*p* > 0.05). Treatment codes as defined in Section 2.2 (Experimental Design).

**Table 3:** Effects of Exogenous Spermidine on the Agronomic Traits of Rice Young Plants under Salt Stress.

Treatment	Plant Height (cm)	Root Length (cm)	Shoot Fresh Weight (mg)	Shoot Dry Weight (mg)	Root Fresh Weight (mg)	Root Dry Weight (mg)
CK	24.29 ± 2.15 ^a^	9.96 ± 2.71 ^ab^	133.63 ± 7.34 ^a^	84.87 ± 6.92 ^a^	24.87 ± 3.67 ^ab^	34.67 ± 2.08 ^bc^
S0	18.39 ± 1.01 ^cd^	7.21 ± 0.58 ^c^	88.24 ± 7.23 ^c^	59.43 ± 6.50 ^c^	14.40 ± 2.16 ^d^	26.33 ± 3.51 ^d^
S1	20.05 ± 1.39 ^bc^	7.46 ± 0.76 ^bc^	105.50 ± 14.55 ^b^	67.67 ± 8.56 ^bc^	20.67 ± 0.58 ^c^	31.33 ± 3.06 ^c^
S2	21.23 ± 0.33 ^b^	9.88 ± 0.55 ^ab^	134.68 ± 5.12 ^a^	75.87 ± 4.77 ^ab^	21.53 ± 0.92 ^bc^	37.67 ± 2.31 ^b^
S3	21.45 ± 0.49 ^b^	10.58 ± 1.32 ^a^	116.23 ± 4.05 ^b^	76.70 ± 1.66 ^ab^	22.53 ± 1.29 ^abc^	43.67 ± 3.51 ^a^
S4	20.77 ± 0.85 ^b^	7.60 ± 1.36 ^bc^	115.77 ± 4.07 ^b^	82.80 ± 5.72 ^a^	26.00 ± 2.00 ^a^	46.33 ± 2.52 ^a^
S5	17.50 ± 0.77 ^d^	7.18 ± 0.77 ^c^	84.87 ± 12.00 ^c^	68.47 ± 3.35 ^bc^	16.10 ± 0.85 ^d^	34.33 ± 1.53 ^bc^

The values in the table are presented as mean ± SD (*n* = 3). Distinct lowercase letters within the same column denote significant differences at *p* < 0.05, while identical letters indicate no significant difference (*p* > 0.05). Treatment codes as defined in Section 2.2 (Experimental Design).

**Table 4:** Principal Component Analysis.

Character	Factor Loading				
	PC1	PC2	PC3	PC4	PC5
PH (m)	0.351	0.091	0.144	0.234	0.081
RL (cm)	0.250	−0.093	−0.106	−0.263	0.651
SFW (mg)	0.312	−0.173	0.298	−0.129	0.167
SDW (mg)	0.325	0.111	0.295	0.233	0.005
RFW (mg)	0.349	0.042	0.199	0.283	−0.174
RDW (mg)	0.259	0.141	0.091	−0.139	−0.556
MDA (μmol/g)	−0.346	−0.057	−0.109	0.062	−0.045
POD (U/g FW)	−0.148	0.461	0.020	−0.178	0.092
CAT (U/g FW)	−0.094	0.430	−0.215	0.236	0.060
SOD (U/g FW)	−0.026	0.396	−0.093	0.544	0.185
SP (mg/g)	0.165	0.391	0.024	−0.392	−0.100
SS (mg/g)	−0.002	0.449	0.208	−0.351	0.169
H2O2 (umol/g)	−0.334	0.064	0.301	−0.084	−0.185
O2-(umol/g)	−0.261	−0.054	0.584	0.000	0.018
O2-PR (umol/min/g)	−0.254	−0.004	0.449	0.188	0.284
Eigenvalue	6.57	3.47	1.48	0.99	0.74
Percentage of Variance (%)	43.81	23.11	9.89	6.59	4.91
Cumulative (%)	43.81	66.92	76.82	83.41	88.32

**Table 5:** Membership Function Analysis of Indicators of the Effects of Exogenous Spd on the Growth and Physiology of Rice Young Plants under Salt Stress.

Treatment	Membership Function					D Value	Rank
	PC1	PC2	PC3	PC4	PC5		
CK	0.949	0.052	0.405	0.494	0.657	0.603	3
S0	0.072	0.19	0.424	0.498	0.699	0.209	7
S1	0.333	0.362	0.252	0.665	0.528	0.367	6
S2	0.439	0.537	0.259	0.591	0.472	0.458	4
S3	0.568	0.877	0.195	0.793	0.565	0.624	2
S4	0.54	0.958	0.535	0.3	0.777	0.644	1
S5	0.331	0.541	0.809	0.413	0.469	0.453	5
*W*_j_	0.496	0.262	0.112	0.075	0.056		

## Data Availability

The original contributions presented in this study are included in the article. The raw RNA-seq datasets generated in this study have been deposited in the NCBI Sequence Read Archive (SRA) under the project accession number PRJNA1355328. Further inquiries can be directed to the corresponding authors.

## Abbreviations

The following abbreviations are used in this manuscript:

CK	control (no NaCl, no spermidine)
S0–S5	75 mM NaCl with 0, 0.6, 0.8, 1.0, 1.2, and 1.4 mM spermidine, respectively
Spd	Spermidine
EBR	Brassinolide
KT	Kinetin
MT	Melatonin
SFW	Shoot Fresh Weight
SDW	Shoot Dry Weight
RFW	Root Fresh Weight
RDW	Root Dry Weight
SP	Soluble Sugar
SS	Soluble Protein
O^2−^PR	O^2−^ Production Rate

## Appendix A

PCR Conditions:

Reaction Mix (20 μL):

10 μL 2× SYBR Green Master Mix (SQ410, Genesand Biotech)

0.4 μL Forward primer (10 μM) and 0.4 μL Reverse primer (10 μM) (Final: 0.2 μM each)

2 μL cDNA (50 ng total RNA)

Nuclease-free water to 20 μL

PCR Cycling Conditions:

Initial denaturation: 95 °C for 30 s

40 cycles of:

Denaturation: 95 °C for 10 s

Annealing: 60 °C for 30 s

Melt curve: 65–95 °C (0.5 °C increments every 5 s)

Note: The annealing temperature can be adjusted based on primer Tm values, usually between 58–62 °C.

**Table A1:** Fluorescent Quantitative Primers.

Gene_ID	Gene Name	Sequences (5′-3′)	
		Forward	Reverse
Os04g0401000	*PI21*	CTGCGATGCCAAGATCAGGAA	CGTACTCCACCTTCTCGATGC
Os03g0760000	*CYP450*	GGGCAACCTGTGCGACTACC	TGTTCCTCACGCCGAACACG
Os02g0626100	*OsPAL1*	GAACGGCAAGCCGGAGTACA	CACCTTCTTGGCGTGGCTCAT
Os03g0246500	*OsPAL*	GACGGAAGCCCTTTCCTTTC	CGAAGAACCGACTGAAACCC
Os08g0489300	*Tag I*	GTGGCGAAGATGGACGAGAA	GTATCCGCTGAACGACCCAA
Os02g0571300	*OsKS5*	GGAGGAATGCGGGAAGCCAA	CTGCACTGGCACCATGGCTA
Os09g0127300	*OsCCR17*	TCCTCCATCGGCACTGTCTA	ATTCGAGGTCACTCCAGCAG
Os07g0603300	*GL7*	CCCTTGCTGCTCTCCAAATC	ACAGCATTAGCAGGGACCTT
LOC4333919	*OsActin*	AGGAAGGCTGGAAGAGGACC	CGGGAAATTGTGAGGGACAT

Note: Gene names corresponding to these locus identifiers are provided when available; not all genes correspond to those shown in Figure 6, as qRT-PCR targets were randomly selected from the DEG dataset.

**Table A2:** Sample Sequencing Quality Analysis.

Samples	Raw Reads	Clean Reads	N/%	Q30/%	GC/%
S0-1	48247616	47612942	0.033	96.30	49.25
S0-2	40536848	40042426	0.032	96.41	49.54
S0-3	44959328	44349634	0.032	96.38	49.39
S1-1	40839448	40361306	0.032	96.29	49.45
S1-2	51109362	50521482	0.033	96.44	49.44
S1-3	36620858	36150452	0.032	96.28	49.70
S4-1	37261728	36731986	0.032	96.13	50.47
S4-2	40284046	39852256	0.033	96.42	49.83
S4-3	44847100	44299712	0.033	96.30	49.77
